# Protein nanoparticles directed cancer imaging and therapy

**DOI:** 10.1186/s40580-021-00293-4

**Published:** 2022-01-08

**Authors:** Yao Miao, Tao Yang, Shuxu Yang, Mingying Yang, Chuanbin Mao

**Affiliations:** 1 School of Materials Science and Engineering, Zhejiang University, Hangzhou, 310027 Zhejiang China; 2 Department of Neurosurgery, Sir Run Run Shaw Hospital, School of Medicine, Zhejiang University, 3 East Qingchun Road, Hangzhou, 310016 Zhejiang China; 3 Institute of Applied Bioresource Research, College of Animal Science, Zhejiang University, Yuhangtang Road 866, Hangzhou, 310058 Zhejiang China; 4 Department of Chemistry & Biochemistry, Stephenson Life Science Research Center, Institute for Biomedical Engineering, Science and Technology, University of Oklahoma, 101 Stephenson Parkway, Norman, OK 73019-5251 USA

**Keywords:** Protein nanoparticles, Nanomedicine, Cancer therapy, Cancer imaging, Virus-like particles

## Abstract

Cancer has been a serious threat to human health. Among drug delivery carriers, protein nanoparticles are unique because of their mild and environmentally friendly preparation methods. They also inherit desired characteristics from natural proteins, such as biocompatibility and biodegradability. Therefore, they have solved some problems inherent to inorganic nanocarriers such as poor biocompatibility. Also, the surface groups and cavity of protein nanoparticles allow for easy surface modification and drug loading. Besides, protein nanoparticles can be combined with inorganic nanoparticles or contrast agents to form multifunctional theranostic platforms. This review introduces representative protein nanoparticles applicable in cancer theranostics, including virus-like particles, albumin nanoparticles, silk protein nanoparticles, and ferritin nanoparticles. It also describes the common methods for preparing them. It then critically analyzes the use of a variety of protein nanoparticles in improved cancer imaging and therapy.

## Introduction

Cancer has threatened the lives of people worldwide for a long time [1]. Late and wrong diagnosis has been the main reason that explains the death of cancer patients [2]. Nowadays, many methods are available for cancer diagnosis, such as electrochemical sensing [2, 3], gas chromatography/mass spectrometry (GC–MS) [4], infrared spectroscopy [5], next-generation sequencing (NGS) [6], immunoassays like enzyme-linked immunosorbent assay (ELISA) and chemiluminescent immunoassay [7], and various biomedical imaging methods [8, 9]. Electrochemical sensors usually diagnose cancer by detecting cancer markers on account of changes in sensor parameters like current or resistance, however, they are sensitive to the environment [10]. Despite the high sensitivity, infrared spectroscopy method usually requires complex instruments. The use of infrared spectroscopy is limited by its relatively low spatial resolution [11]. NGS can realize simultaneous sequencing of multiple copies for tumor genomic assessment, but the data analysis is very complicated [6]. Although immunoassays are sensitive and selective, they are limited by long time consumption and high cost [7]. Biomedical imaging has been widely used in cancer diagnosis due to their advantages such as intuitional real-time imaging and minimal or no invasiveness [12]. Also, biomedical imaging can be easily combined with targeted cancer therapy [13].

Besides cancer cells, the tumor microenvironment provides convenience for the occurrence and development of tumors [14]. Distorted blood vessels, high interstitial pressure, hypoxia, and the possibility of metastasis all make tumor treatment more difficult [15–18]. At present, the main treatment of tumors is surgical resection, supplemented by chemotherapy and radiotherapy, and the clinical practice uses chemotherapy in many areas because of its convenience [19, 20]. However, single chemotherapy has many limitations. Multidrug resistance (MDR) is one of the primary reasons for failure in tumor chemotherapy [21]. Although cancer cells are more sensitive to chemotherapeutic drugs than normal cells, the toxicity of chemotherapeutic drugs to normal cells and organs like the liver and spleen can cause serious side effects at therapeutic doses [19, 22]. Also, many anti-cancer drugs are insoluble or slightly soluble in water, which increases the difficulty of drug delivery [23]. In order to solve these problems, it’s necessary to use emerging nanotechnology to construct novel nanocarriers and corresponding drug delivery systems to achieve combination therapy.

Current nanocarriers include inorganic nanoparticles such as iron oxide, quantum dots, gold nanoparticles, and organic nanoparticles such as liposomes and dendritic polymers [24–28]. Although inorganic nanoparticles have been used widely to treat cancer because of their unique optical properties or potential magnetic and catalytic properties, some of them face the problems of in vivo long-term circulation and potential toxicity [29, 30]. Liposomes have good biocompatibility, but they are easily oxidized, structurally unstable, and prone to drug leakage [30]. In order to enhance their therapeutic effects, it is necessary to design nanoparticles that can release a drug in response to stimulation, which undoubtedly complicates the inherently sophisticated synthesis process of nanoparticles [31].

**Fig. 1:**
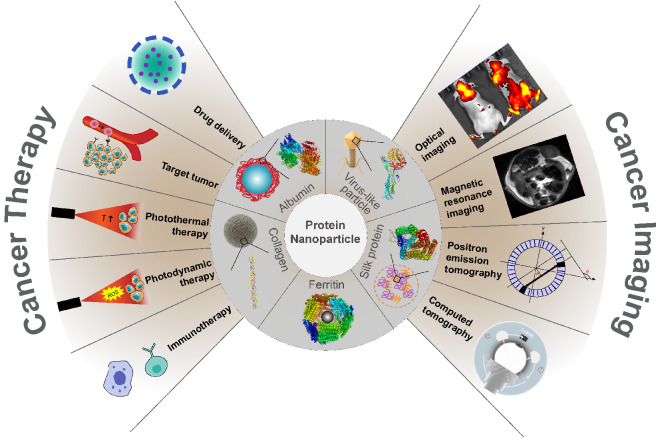
The schematic illustration of protein nanoparticles directed cancer imaging and therapy

Protein nanoparticles stand out among nanocarriers because of their biocompatibility, biodegradability, and low immunogenicity. Also, their preparation conditions are mild, with no need for toxic solvents [32]. One of the FDA-approved drugs for clinically treating metastatic breast cancer, Abraxane ®, is albumin-based nanoparticles loaded with paclitaxel. The drug can inhibit tumor growth by suppressing the mitosis of breast cancer cells, and the use of human’s most abundant protein albumin can avoid the hypersensitivity caused by previous drug solvents such as Cremophor EL [33]. The surface of protein nanoparticles can be easily modified due to the presence of surface functional groups on them [34]. The hollow structure of some proteins allows for the convenient loading of small molecule drugs or metal nanoparticles for drug delivery and combination therapy [35–37]. This review mainly talks about the design and preparation of protein nanoparticles and their theranostic use in cancer imaging and therapy (Fig. 1).

## Design of protein nanoparticles

### Types of protein nanoparticles

#### Virus-like particles

Virus-like particles (VLPs) are composed of self-assembled viral coat proteins. They have structures highly similar to natural viruses, but they have no ability to self-replicate in human cells due to the lack of viral nucleic acid [38]. Their particle size ranges from tens of nanometers to hundreds of nanometers, including animal viruses, plant viruses, and phages [39–42]. VLPs’ viral nature makes them perfect carriers for therapeutic nucleic acids [43], and the hollow structure allows them to load other types of molecules such as drugs [35], siRNA [44], antibodies [45], and nanoparticles [36]. VLPs are uniform in shape and easy to functionalize, giving them a huge advantage in drug loading and cancer therapy [46]. The methods for surface modification of VLPs can be chemical or genetic. Residues of natural amino acids can be covalently linked through chemical couplings, such as the sulfhydryl of cysteine, the amine of lysine, and the carboxyl of aspartate or glutamate. Unnatural amino acids like azidohomoalanine and p-amino-phenylalanine have also been used to modify VLPs through global methionine replacement and amber stop codon suppression [47]. In addition, foreign antigens or any other surface ligands can be genetically fused into the VLPs’ coat proteins to form chimeric VLPs [48, 49].

Some VLPs can be highly immunogenic, so they can be used as vaccines for safe tumor immunotherapy [50]. The reason why VLPs can elicit strong immune responses is that the unique structure of microbial antigens is able to make mammals respond vigorously towards them through inducing humoral and cell-mediated immunity [51]. What’s more, immunocompetence varies according to the types of VLPs [48]. VLPs mainly accumulate in the liver and spleen of mice after intravenous injection. Farkas and his coworkers injected bacteriophage MS_2_ and PEGylated MS_2_ into mice to study their biodistribution and the effect of PEGylation. Both MS_2_ and PEGylated MS_2_ labeled with ^64^Cu were mainly distributed in the liver and spleen. However, PEGylated MS_2_ showed significantly reduced accumulation in the spleen as it avoided the immune system [52]. Other VLPs like CCMV and CPMV behave similarly [47].

#### Albumin

Albumin is thought of as one of the most abundant proteins in blood vessels. It includes ovalbumin (OVA), bovine serum albumin (BSA), and human serum albumin (HSA) [53, 54]. OVA is a kind of food protein whose molecular weight is 47 kDa. It is both pH-sensitive and temperature-sensitive [55]. BSA’s molecular weight is 69.323 kDa [56]. HSA’s molecular weight is 66.5 kDa [57]. Unlike OVA, HSA is stable in organic solvents and not sensitive to pH and temperature.

Albumin allows for a long-time circulation in the blood, and is biodegradable and soluble. The circulating half-life of human albumin is up to 19 days [58]. Being soluble in water and bearing diverse drug binding sites make albumin an ideal candidate for drug carrier, especially for drugs with poor solubility [59]. Protein nanoparticles made of albumin inherit the advantages of albumin such as biodegradability. Their functional groups on the surface make it easy for surface modification and linking with drugs covalently [60]. Surface modification of albumin nanoparticles can be achieved through the chemical coupling of covalent bonds or non-covalent interactions, such as hydrophobic and electrostatic interactions. The amino, thiol, and carboxyl groups on the surface of albumin make it easy to graft ligands and load drugs through chemical reactions [53, 61]. Sepehri et al. constructed conjugates of HSA and 7-ethyl-10-hydroxy-camptothecin (a kind of cancer drug) through the N-(3-Dimethylaminopropyl)-N’-ethylcarbodiimide hydrochloride (EDC) and N-hydroxysuccinimide (NHS) reaction. HSA improved the solubility and pharmacokinetics characteristics of the drug [62]. In addition, albumin nanoparticles can be modified by surfactants [63].

Albumin will accumulate in the liver and bladder after intravenous injection [64]. Zhang et al. [65] studied the pharmacokinetics and biodistribution of bufalin-loaded BSA nanoparticles. The results showed that BSA modification prolonged the half-life of bufalin (cancer drug) by more than 6 h, reduced its clearance, and made it more easily ingested by the liver.

#### Silk proteins

Natural silk proteins usually include silk fibroin and sericin from silkworm cocoons and spider silk protein [66]. As a major protein, silk fibroin accounts for more than 65% of total silk proteins [67]. Silk fibroin has many hydrophobic domains and interval hydrophilic domains, and is mainly composed of glycine, alanine and serine [68]. The anti-parallel β-sheets of silk fibroin’s heavy chain endow the fiber stability and mechanical properties. The advantages such as biocompatibility, biodegradability and low immunogenicity make silk fibroin useful in drug delivery [69]. Silk sericin is what wraps silk fibroin fibers and is rich in hydrophilic groups [70]. Different from silk derived from silkworm, spider silk has no sericin coating and barely elicits immunological reaction [71].

Silk protein nanoparticles exhibit high affinity with drugs, promote controlled drug release (pH-dependent), and can be prepared facilely, making them strong candidates as drug carriers for cancer therapy [72, 73]. Silk protein nanoparticles can be modified with drugs or ligands through covalent bonding such as EDC/NHS reaction or non-covalent linking like physical adsorption and coprecipitation [74, 75]. Silk protein nanoparticles can be used to encapsulate hydrophobic drugs. In addition to small molecule drugs, silk protein nanoparticles can also deliver proteins, peptides, and genes [72]. It is worth mentioning that electronegative silk protein nanoparticles can be degraded by proteolytic enzymes, so the most likely way of metabolism is absorption by the body [76].

#### Collagen

Collagen is a type of fibrin widely spread in various tissues of the body, comprising 30% of all kinds of proteins. It is the most abundant protein in animals [77]. There are more than 30 types of collagens, and every collagen type contains three helix polypeptide chains. In addition, collagen is an important component of the extracellular matrix [78]. Biodegradability, biocompatibility, and weak antigenicity are collagen’s representative properties, and the low immunogenicity can be eliminated by heat or chemical treatment [79]. Collagen’s molecular weight is 300 kDa. It turns into gelatin after irreversible hydrolysis. Collagen-based nanoparticles can decrease the systemic toxicity of drugs, enhance the uptake of nanoparticles by cells, and also are thermally stable [80].

#### Ferritin

Ferritin can store iron and has a hollow cavity. It is made up of a spherical polypeptide shell (apoferritin) and a hydrated iron oxide core (5Fe_2_O_3_∙9H_2_O) with a diameter of 6 nm [81, 82]. Therapeutic agents can be encapsulated within ferritin by reversible self-assembly. There are two kinds of ferritins, heavy (H) and light (L), and they can complement each other. Each ferritin is made up of 24 subunits. Despite a rigid structure of ferritin, the connection between its 24 subunits is pH-dependent, indicating that it decomposes under acidic conditions and re-assembles itself in a neutral environment [83]. Unlike other temperature-sensitive proteins, ferritin is thermostable due to hydrogen bonds and salt bridges between subunits [84].

The binding of ferritin to transferrin receptors allows it to be internalized by tumor tissues, and thus ferritin nanoparticles can be applied to deliver drugs. In addition, the surface groups of ferritin like amino, carboxyl, and sulfhydryl groups can be connected with ligands chemically, and its cavity can bind with metals with high affinity, making it a multifunctional nanocarrier for effective drug delivery [27, 85, 86]. PEG can be used to modify the surface of ferritin and the PEGylated ferritin can circulate longer in vivo [87]. Ferritin has a strongly negative charge in its inner cavity, so it can be easily bonded with positively charged metal ions by electrostatic interactions [88]. What’s more, ferritin can also be biologically modified by genetic recombination techniques [89].

Beeman and his coworkers injected cationized ferritin into healthy rats intravenously using ferritin itself as a contrast agent for magnetic resonance imaging to study its biodistribution. The results showed that ferritin was distributed at the kidney, liver, lung, and spleen and could be cleared by the liver after 7 days. Furthermore, ferritin had no toxic effects on the kidney and liver [90]. A summary and comparison of the aforementioned protein nanoparticles (PNs) are concluded in Table 1.

**Table 1:** Summary and comparison of protein nanoparticles (PNs)

Types of PNs	Classification	Characteristics	Refs.
Virus-like particles	Animal viruses, plant viruses and phages	Highly immunogenic, hollow structure	[35, 50]
Albumin	OVA, BSA, HSA	Long-time circulation, diverse hydrophobic binding sites	[59, 65]
Silk proteins	Silk fibroin and sericin	Certain mechanical strength, electronegative, pH-responsive drug release	[69, 73, 76]
Collagen	More than 30 types	The most abundant protein	[77]
Ferritin	heavy (H) and light (L)	pH-dependent self-assembly, thermostable, imaging	[83, 84, 90]

### Preparation strategies of protein nanoparticles

#### Desolvation

The desolvation method is also called the coacervation method and is commonly used in the preparation of protein nanoparticles [72]. Desolvation refers to adding a reagent that can reduce the solubility of the protein in the protein solution so that the protein can agglomerate and settle. The different solubility of proteins in different solvents is exploited to realize phase separation [32]. The size of protein nanoparticles can be controlled by multiple variables, such as protein concentrations, ratios of desolvating agent (antisolvents) to original solvents, and pH values. Higher pH values tend to produce nanoparticles with smaller sizes. After the formation of protein nanoparticles, cross-linking agents like glutaraldehyde are added [91, 92]. The advantages of the desolvation method include relatively mild reaction conditions, no need for surfactants, and a time-saving simple preparation process [72]. This method is widely used in the drug encapsulation of protein nanoparticles [93]. For example, Weber et al. [34] prepared albumin nanoparticles through the desolvation method. They explored the influence of various process variables on the surface amino groups and particle size of nanoparticles. They found out that desolvation had a great effect on the size of nanoparticles.

Matrix density is a vital factor to influence the drug release rate for protein nanoparticles used in drug loading. Rao et al. synthesized gelatin nanoparticles with different matrix densities by the desolvation process [94]. They investigated the effect of pH and desolvating agent concentrations on gelatin nanoparticle synthesis in detail and concluded the mechanism. By changing the pH, they could vary the size and matrix density of gelatin nanoparticles easily. Then, the resistance to protease and fluorescein release was compared among gelatin nanoparticles with different matrix densities. The results indicated the potential of protein nanoparticles synthesized by the desolvation method in regulating the drug release rate [94].

#### Emulsification

Emulsification is a method to prepare nanoparticles through the formation of emulsion. Emulsion is a multi-phase dispersion system formed by two immiscible liquids. The liquid in a droplet form is named the dispersed phase, and the other is named the continuous phase [95]. The basic types of emulsions include oil-in-water (O/W) and water-in-oil (W/O). Surfactants have an important influence on the process of emulsification to form an emulsion [96]. In the emulsion constituted by oil and water, the phase with a high surfactant solubility is generally the continuous phase of the emulsion. After emulsification, flocculation, coalescence, and Ostwald ripening usually happen during the aging of emulsion as it is a thermodynamically unstable system [95]. For the preparation of protein nanoparticles, emulsions usually form by mixing an aqueous phase containing the protein and an organic phase containing surfactants with a miscible solvent of oil and water [97]. Then the protein nanoparticles will be generated by adding such emulsions to preheated or precooled oil drop by drop. Wu et al. developed a novel PEG-assisted emulsification process to prepare silk nanoparticles for oral delivery of curcumin [98]. The liquid PEG with a low molecular weight was used as the organic phase, which was green and safe in the application of delivering drugs. The nanoparticle size could be controlled easily by tuning the concentration of silk protein. The β-sheet structures of silk protein could form strong hydrophobic interactions with the phenol groups of curcumin [98]. It helped the nanoparticles to get an efficient drug loading and great ability to control cargo release.

**Fig. 2:**
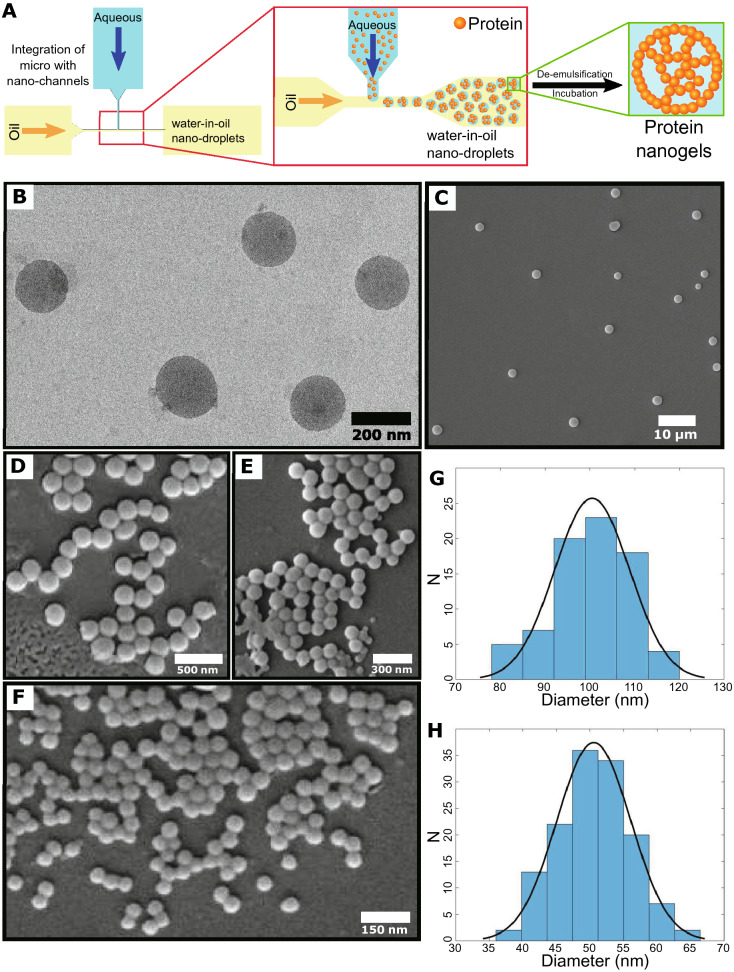
Protein nanoparticles preparation through an emulsification strategy with integration of microfluidics/nanofluidics technology. **A** Illustration of protein nanogels formation by the production of monodisperse water-in-oil nanodroplets in the nanochannels and subsequent de-emulsification steps. **B** Transmission electron microscopy (TEM) and **C**–**F** Scanning electron microscopy (SEM) analysis of silk fibroin nanoparticles. **G**, **H** are the size distributions of nanoparticles in (**E**) and (**F**), respectively [99]

The emulsion method is also used in combination with other advanced technology platforms to prepare protein nanoparticles. By integrating emulsification with the microfluidics/nanofluidics technology, Toprakcioglu et al. [99] described a protein nanogel preparation strategy for intracellular delivery vehicles. Monodisperse nanodroplets were the key to the W/O emulsion formation. They fabricated the nanofluidic device using a two-step lithographic method for generating the droplets. Nanoemulsions production can be tuned by the relationship between the current speed of the dispersed phase and the continuous phase. After the collection of protein nanodroplets, a de-emulsified and subsequently re-emulsified process could be done to get the aqueous phase protein nanogel [99]. Electron microscopy results indicated precise control of emulsion sizes and a narrow distribution with the dispersion (Fig. 2). They could use this approach to generate silk, β-lactoglobulin, and lysozyme nanoparticles, verifying the versatility of this strategy.

#### Salting out

Salting-out is a very simple but efficient way to prepare protein-based nanoparticles. The process is a bit like desolvation, but no organic solvents are included. Protein coacervates will form from solutions by adding salt ions with a relatively high concentration [67, 100]. Since salt ions are more hydrophilic than protein, they compete with protein micelles and are combined with water, destroying the protein hydration layers. This decreases the solubility of the protein. In addition, the salts for the salting-out method have a strong dissociation effect. The dissociation of salt can inhibit the dissociation of weak protein electrolytes, which reduces the charge of the protein and makes it easier to aggregate and precipitate [72, 101]. The conformational structure of proteins stays the same after the salting-out based strategy. So, the bioactivity and function of proteins can be retained after the nanoparticles are formed. Silk fibroin nanoparticles with controlling features were prepared by the salting-out process for drug delivery [102]. The morphology and salting-out efficiency were determined by the pH value as well as the ionic strengths of potassium phosphate solutions. The drawback of the salting-out method is the wide size distribution of protein nanoparticles by the approach.

#### Spray drying

Spray drying is a liquid atomization technology, an important method for producing a drying powder. It disperses the solution into small droplets through a nozzle, transmits heat and mass using a hot and drying medium, and obtains dry particles through the evaporation of the solvent [103–105]. Recently, spray drying has been well-established as the preparation strategy for protein nanoparticles [106, 107]. Most of the protein nanoparticles produced by the spray drying are spherical, with good fluidity and dispersibility. Tan et al. found a simple nano spray drying method for improving collection efficiency of a typical spray drying method [107]. BSA nanoparticles were produced through the newly designed Nano Spray Dryer B-90. The size and morphology of nanoparticles mainly depend on the surfactant concentrations and spray mesh sizes. Spray drying granulation has the advantages of continuous work, convenient operation and control, and is suitable for large-scale production [104, 106].

#### Self-assembly

**Fig. 3:**
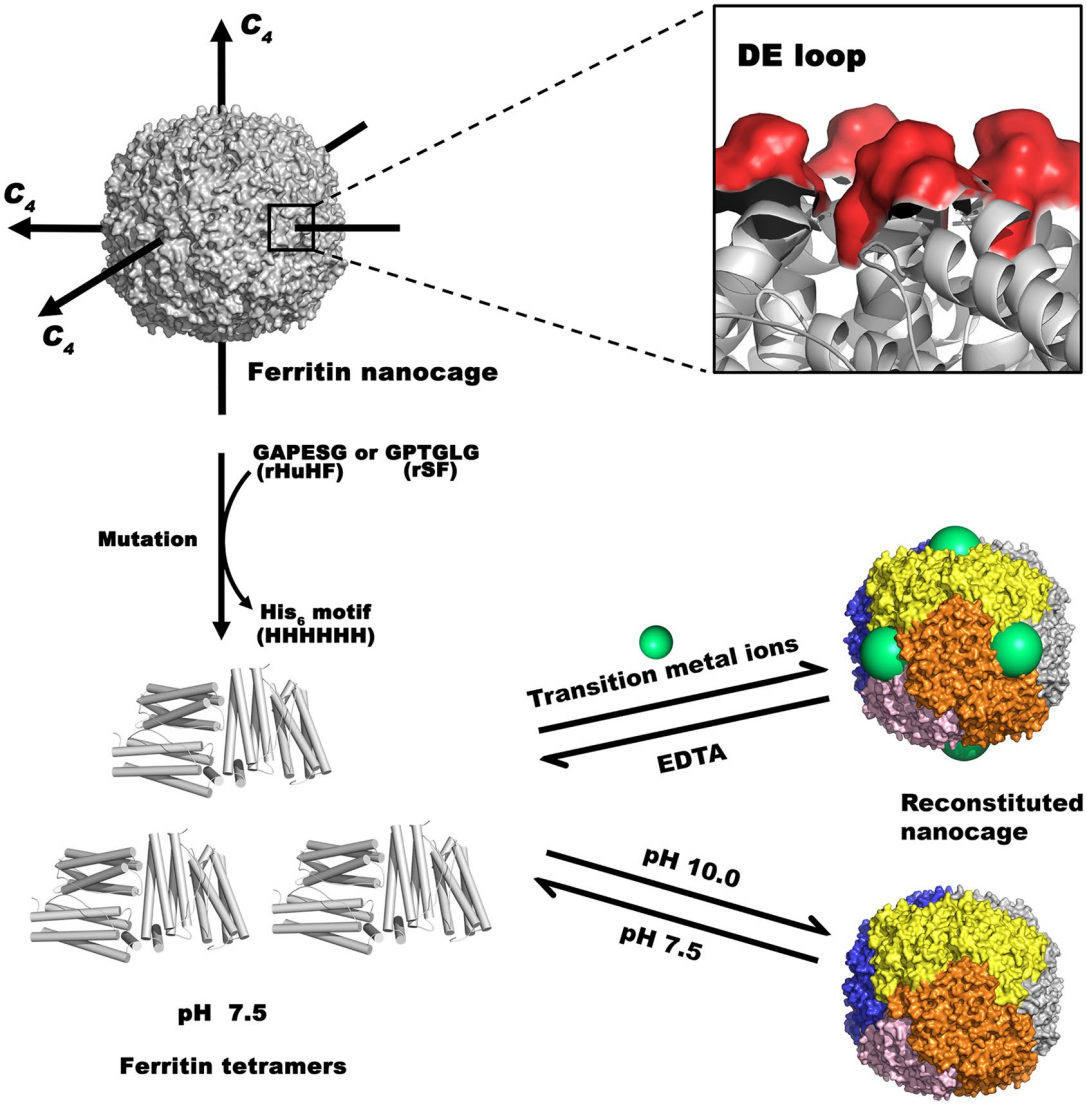
An example of designing and preparing protein nanoparticles by self-assembly. Six amino acids were mutated to HHHHHH (His_6_) located in the DE loop of ferritin proteins. The resultant subunits were natural ferritin tetramers at pH 7.5. Such tetramers could self-assemble into reconstituted nanocages at pH 10.0 or by the induction of transition metal ions at pH 7.5. Conversely, the nanocages would be disassembled into ferritin tetramers by the stimuli of pH or EDTA [112]

Since protein is a natural amphiphilic polymer, it has the potential to form nanoparticles through self-assembly. More and more protein supramolecular nano-complex systems are formed by self-assembly of naturally or artificially prepared protein subunits through non-covalent bonds [108, 109]. Ferritin is a rigid structure under neutral pH conditions. When the pH is dropped to 2, the ferritin nanocage structure is destroyed and disassembled to form a single subunit. When the pH of the system is adjusted to neutral again, the ferritin subunits can re-assemble to form a nearly complete structure [110, 111]. This unique property makes self-assembly an ideal strategy for encapsulating bioactive compounds into ferritin nanoparticles. In a recent study, the self-assembly of ferritin nanocages was regulated by introducing His motifs through genetic engineering [112]. When the solution pH equaled to 7.5, ferritin proteins were assembled into natural tetramers. If the pH was adjusted to 10.0 or by adding Ni^2+^, ferritin tetramers would self-assemble into reconstituted ferritin nanocages. This process was reversible as pH changed back to 7.5 or under the stimuli of EDTA (Fig. 3).

It is normal to obtain VLPs using self-assembly [113, 114]. Hong et al. used the self-assembly method to prepare VLPs originating from bacteriophage P22 [115]. The model antigen peptide was displayed onto the coat protein (CP), which was then self-assembled with the scaffolding protein (SP) to get the P22 VLP-Antigen Peptide with an icosahedral structure. Further results verified that the VLP was useful to work as a platform to deliver peptide antigens with high efficiency in therapeutic cancer vaccines. A comparison of the advantages and disadvantages of different preparation strategies of protein nanoparticles is summarized in Table 2.

**Table 2:** Comparison of advantages and disadvantages of common synthesis methods of protein nanoparticles

Preparation strategy	Advantages	Disadvantages	Key factors
Desolvation	Mild conditions, without surfactants, and simple process [72]	Unstable particle size [34]	pH [72]
Emulsification	Uniform size [99]	Necessary surfactants, complex preparation process [96, 97]	Monodisperse droplets [99]
Salting out	Simple and green preparation [67]	Wide size distribution [102]	pH [102]
Spray drying	Good particle dispersion, convenient operation and control, and suitable for mass production [104, 106]	Equipment support required [107]	Surfactant concentrations and spray mesh sizes [107]
Self-assembly	Reversible [110]	Limited scope of application	pH [112]

### Advantages of protein nanoparticles for cancer nanomedicine

Ideally, the use of nanoparticle platforms for oncological applications should be sufficiently effective to produce a unique function in vivo with minimal toxicity and low risk to the body [116, 117]. Noble metal-derived nanoparticles (platinum, gold, etc.) have inherent therapeutic characteristics due to their unique optical and electronic properties. However, the unknown long-term accumulations in the host body have limited their clinical considerations [116, 118]. Inorganic nanoparticles can be easily synthesized and modified, but they may release excess toxic ions when used for cancer treatment [119, 120]. For instance, carbon nanomaterials are usually hard to degrade in vivo and have the risk of increased side effects [121, 122]. Polymeric nanoparticles possess good biocompatibility and manufacturing advantages, but they have limited tumor targeting ability without additional ligand modifications [117, 123]. Compared with other nano-systems for cancer therapy and imaging, protein-based nanoparticles have several unique advantages: (1) Proteins can be produced through bioengineering strategies, avoiding the use of chemical synthesis and toxic solvents [67, 124]. (2) Given the numerous epitopes and microstructures on the surface of proteins, the modification and production for improving the function of nanoparticles are relatively easy. Especially, a genetic engineering strategy can be used to display antigen epitopes or other functional groups on the protein nanoparticles for cancer nanomedicine [115, 125–127]. (3) Protein is naturally amphipathic, enabling the hydrophobic domain of proteins to interact with lots of hydrophobic anti-tumor drugs for improving the drug encapsulation capability of nanoparticles. (4) Some proteins have a natural tropism for tumors, and other targeted ligands can also be modified onto the surface of the protein nanoparticles for targeting tumors [128, 129]. (5) Due to the nontoxicity and high drug encapsulation capacity, protein-based nanoparticles used for drug cargo loading can achieve a higher intratumor drug concentration [124]. (6) The metabolic products of protein nanoparticles are amino acids, which are non-toxic and harmless to humans.

## Cancer therapy by using protein nanoparticles

### Drug cargo loading

A lot of protein nanoparticles have been developed as ideal drug cargo loading agents for cancer therapy to reduce drug toxicity and improve therapeutic effects. Compared with normal drug delivery systems such as inorganic nanoparticles, protein nanoparticles have incomparable advantages such as biocompatibility and biodegradability [32]. The albumin's affinity with hydrophobic drugs is reversible, and the complex is allowed to be transported in the blood and released on the specific cell surface [130, 131]. In cancer therapy, the first protein nanoparticle approved by FDA as a drug carrier for clinical use is the albumin combined with paclitaxel (Abraxane®) for the treatment of metastatic breast cancer [124]. Also, researchers constructed hyaluronic acid (HA) coated albumin nanoparticles and used the composite nanoparticles to load both hydrophobic and hydrophilic drugs due to the hydrophobic binding sites of albumin as well as the hydrophilic binding sites of HA [93]. The binding strength between HSA and drug is significant because it determines when the drug is released in vivo. Qi et al. [132] took advantage of the hydrophobic cavity of HSA IIA subdomain, and used it to combine ferric anti-cancer agents. The non-immunogenicity, low cytotoxicity, and multiple drug binding sites give HSA nanoparticles a big advantage over other drug loading systems. HSA-based nanoparticles can also avoid unnecessary interactions with sera in addition to increasing the solubility of hydrophobic molecules in the blood [133].

In addition, protein nanoparticles can use their hollow structures to load drugs. Auoxo3, a gold compound, is a kind of cytotoxic anti-cancer drug. Ferraro et al. [134] used a ferritin nanocage to encapsulate it. The results of cell experiments showed that compared to non-tumorigenic cells, the nanocarriers were much more toxic to aggressive human cancer cells. Also, Huang et al. [135] used the H-ferritin’s cavity to load anti-cancer drug (doxorubicin, DOX) through pH-mediated decomposition and re-self-assembly, achieving a high load of up to 458 DOX molecules per nanocage. Although inorganic porous nanomaterials can also be used to deliver drugs, some of them even have a high drug loading rate. However, without modification, they cannot avoid drug leakage during transportation [136]. The unique cavity structure and pH-dependent self-assembly and decomposition of ferritin give it unique superiority in drug delivery. The combination of disulfiram and copper is believed to have anti-tumor properties. Xu et al. [137] firstly linked albumin (HSA) to ferritin (Fn) chemically and used the cavity of ferritin to encapsulate Cu^2+^. Together with disulfiram, the Fn-HSA-Cu complex showed a great antitumor ability both in cell and animal experiments.

Wang and his coworkers [138] took advantage of silk protein’s negative surface charge and combined the silk protein with silica into nanocomposites for drug delivery. Both bombyx mori silk fibroin (SF) and sericin (SS) have a negatively charged surface and a high encapsulation efficacy. Therefore, the silk proteins were used as biotemplates to direct the self-assembly of silica nanoparticles and nanofibers. The nanocomposites were employed to load DOX, and the drug loading capacity could be as high as 33% after mixing for 24 h, much higher than other silica nanoparticle-based drug loading systems. Analogously, Shuai et al. [139] used wild silkworm’s sericin as a template to induce nucleation of hydroxyapatite (HAp) nanoneedles. The porous sericin-HAp nanocomposite microspheres could adsorb DOX through electrostatic adsorption and achieved controllable release of DOX.

Collagen is a kind of fibrous protein available in the extracellular matrix of different tissues, including tumors [140, 141]. As a nature-derived biopolymer, collagen is widely used as the matrix material for drug cargo loading [142]. Protein-based compositions can enhance the solubility of the whole nano-systems. The fibrous structural characteristics of collagen can increase the mechanical stability of nanoparticles [117, 142]. Jiang et al. [143] used poly (3-acrylamidophenylboronic acid) to bind collagen to build collagen-based nanoparticles (collagen-PAPBA). They used the nanoparticles to envelop DOX and the encapsulation efficiency was up to 97%. The in vitro results showed that DOX-loaded collagen-PAPBA had significant cytotoxicity towards ovarian cancer cells. In another study, collagen was combined with nanohydroxyapatite and fucoidan to prepare nanomaterials for drug delivery [142]. The physico-chemical properties of the materials were improved with better crosslinking and non-toxicity to overcome the original disadvantages. A cell migration assay indicated that the composite material is a promising drug delivery platform for killing cancer cells.

### Tumor targeting

In the past, many tumor-targeting drugs relied on passive targeting based on the enhanced permeability and retention (EPR) effect [144]. Nanodrugs or nanoparticles can target tumors actively through the affinity with highly expressed tumor-specific receptors in tumor cells or tumor microenvironment such as tumor blood vessels and fibroblasts [145].

SPARC and gp60 are highly expressed in glioma and tumor vascular endothelial cells. They can bind specifically to albumin. Lin et al. [146] developed self-assembled albumin nanoparticles, which encapsulated two kinds of hydrophobic anti-cancer drugs, paclitaxel, and fenretinide. The composite nanoparticles could target in situ and subcutaneous glioma effectively. The cell-penetrating peptide (CPP) LMWP modified on the nanoparticles enhanced the interaction between the nanoparticles and cancer cells, leading to better targeted delivery and intensive tumor accumulation.

Li et al. [147] developed a ZnF_16_Pc loaded ferritin nanocage. ZnF_16_Pc is a kind of near-infrared photosensitizer. They then attached an affinity single-chain variable fragment (scFv) of fibroblast activation protein (FAP) to the surface of nanoparticles. Since FAP was over expressed in over 90% cancer-associated fibroblasts, the results showed that nanoparticles could target 4T1 breast cancer subcutaneous tumor. In addition, they found that photodynamic therapy increased particle aggregation at the tumor site.

In addition to the binding with specific and highly expressed receptors in the tumor microenvironment, protein nanoparticles can actively target tumors by grafting targeting peptides on the surface as well. Integrin is overexpressed on many tumor cells. Bari et al. [148] prepared silk fibroin nanoparticles and modified cyclopentapeptides (cRGDs) on their surface, which had a high affinity for integrin αvβ3 and αvβ5. The targeting peptide significantly increased the particle internalization into human urinary bladder (ECV) cancer cells. Shan et al. [149] modified the tumor-targeting peptide RGD onto the surface of hepatitis B core protein (HBc) based VLP genetically. HBc-VLP was found to target tumors through the binding of RGD with integrin αvβ3 overexpressed in B16F10 and U87MG cancer cells. VLPs inherit the advantages of proteins and can be genetically modified with targeting peptides, whereas inorganic nanoparticles are generally modified with the targeting ligands through direct chemical coupling or through the use of organic polymer linkers [150]. In addition, studies have shown that the targeting ability of VLPs will not be adversely affected by protein corona [151]. However, when silica was modified with transferrin, the protein corona formed on the surface of silica would shield transferrin, making it lose the ability to bind with transferrin receptors. They were highly expressed in many tumors, thus losing the ability to target tumors [152].

### Stimuli-responsive releasing

Protein nanoparticles can deliver drugs responding to internal stimuli such as glutathione (GSH), pH value, and enzyme concentration or external stimuli like temperature through engineering [153]. Seib et al. [73] prepared silk protein nanoparticles by an acetone precipitation method. The produced silk nanoparticles were negatively charged, and hence they could be loaded with positively-charged DOX through electrostatic attraction. When composite particles entered the lysosome (pH = 4.5), silk protein lost negative net charge on its surface so as to release drugs, realizing pH-responsive drug release without any chemical modification. The research indicated that silk protein nanoparticles had the potential to become a powerful delivery platform for lysosomal drugs. Pancreatic cancer is malignant and is closely related to the nervous microenvironment. Lei et al. [154] used ferritin nanocages to encapsulate two drugs respectively to explore the effect of neuro microenvironment on pancreatic cancer, including carbachol to activate neural activity (Nano-Cab NPs) and atropine to weaken neural activity (Nano-Ato NPs). Both of them could target pancreatic tumors via transferrin receptors. Because of the nature of ferritin, drugs would be released in the slightly acidic tumor microenvironment rather than in the neutral blood or alkalescent pancreatic juice. A mutant of Hepatitis B core antigen (tHBcAg) can form icosahedral VLP through self-assembly. Biabanikhankahdani et al. [155] mixed positively charged DOX with negatively charged polyacrylic acid (PAA) and loaded them into tHBcAg VLP. The electrostatic attraction between DOX and PAA was reversible when the pH was below 5.5, thus achieving the pH-responsive release of DOX.

Gou et al. [156] developed a multi-responsive drug delivery vehicle with regenerated silk fibroin (RSF). The results confirmed that RSF nanoparticles responded to acidity, ROS, GSH, or hyaluronidase on account of the destroy of β-sheet structure and cleavage of disulfide bonds, leading to accelerated drug release. Thong et al. [157] developed a kind of *Macrobrachium rosenbergii* nodavirus-based VLP (MrNVLP), and DOX was encapsulated inside through an infusion method. This thermally responsive nanocarrier was able to release DOX much faster at 43℃ than 37℃ because MrNVLP had a more relaxed structure at a higher temperature.

### Photothermal therapy (PTT) and photodynamic therapy (PDT)

Both photothermal and photodynamic therapy are light-mediated therapy [158]. Photothermal therapy generally uses nanomaterials themselves or photothermal agents to transform the absorbed light energy into heat energy to raise the temperature of a tumor to kill cancer cells. Apoferritin (AFN) is a pH-sensitive spherical cage protein. He et al. [159] used this protein nanoparticle as a drug carrier to carry the anti-cancer drug paclitaxel (PTX), and then conjugated the second near-infrared region dye IR1061 and folic acid onto protein nanoparticles through a chemical reaction. Nanoparticles can target tumors and become internalized by cancer cells due to the overexpressed folate receptors on their membrane. After intravenous injection of the composite nanoparticles into breast cancer tumor-bearing mice, the temperature of tumor sites increased significantly with 1064 nm laser irradiation and thus inhibited the tumor growth through photothermal-chemotherapy. The thermal stability of ferritin makes ferritin nanoparticles competitive nanocarriers for photothermal cancer therapy. Wang et al. [160] used negatively charged silk fibroin (SF) as a template to direct the self-assembly of positively charged gold nanoparticles (AuNPs). The resultant AuNPs/SF nanofibers were proved to be effective photosensitizers for PTT. After in-situ injection, the nanofibers could inhibit tumor growth effectively when the tumor was exposed to 808 nm laser irradiation, while simple gold nanoparticles couldn’t. Xu et al. [161] used self-assembled SF protein nanoparticles to encapsulate a photothermal agent ICG dye. The composite particles inhibited the growth of xenograft glioblastoma by PTT, realizing the integration of diagnosis and treatment.

Photodynamic therapy is a kind of oxidative therapy. Photosensitizers are employed to transfer light energy to chemical energy and turn the surrounding oxygen molecules to highly active singlet oxygen (^1^O_2_) to kill cancer cells [162, 163]. Wang et al. [164] designed a BSA-based hypoxia-responsive nanoparticle. Photosensitizer Ce6 was covalently connected to BSA, and together with ferritin and ferroptosis-inducing drug sorafenib (SRF) they formed a nanoreactor. In addition to ^1^O_2_ produced by photosensitizers through photodynamic therapy, ferritin-induced Fenton reaction also produced hydroxyl radicals to kill cancer cells. Also, SRF inhibited the synthesis of GSH, decreased the expression of glutathione peroxidase 4 (GPX4), accelerated the lipid peroxidation, thus finally inducing ferroptosis. Rhee et al. [165] modified icosahedral bacteriophage Qβ VLPs with a metalloporphyrin derivative, which was a kind of photosensitizer, and glycan, which could target the CD22 receptor. They co-incubated the VLPs and CHO cells to verify the photoactivity of VLPs in vitro. The results showed that these VLPs were more cytotoxic to CD22 positive CHO cells.

**Fig. 4:**
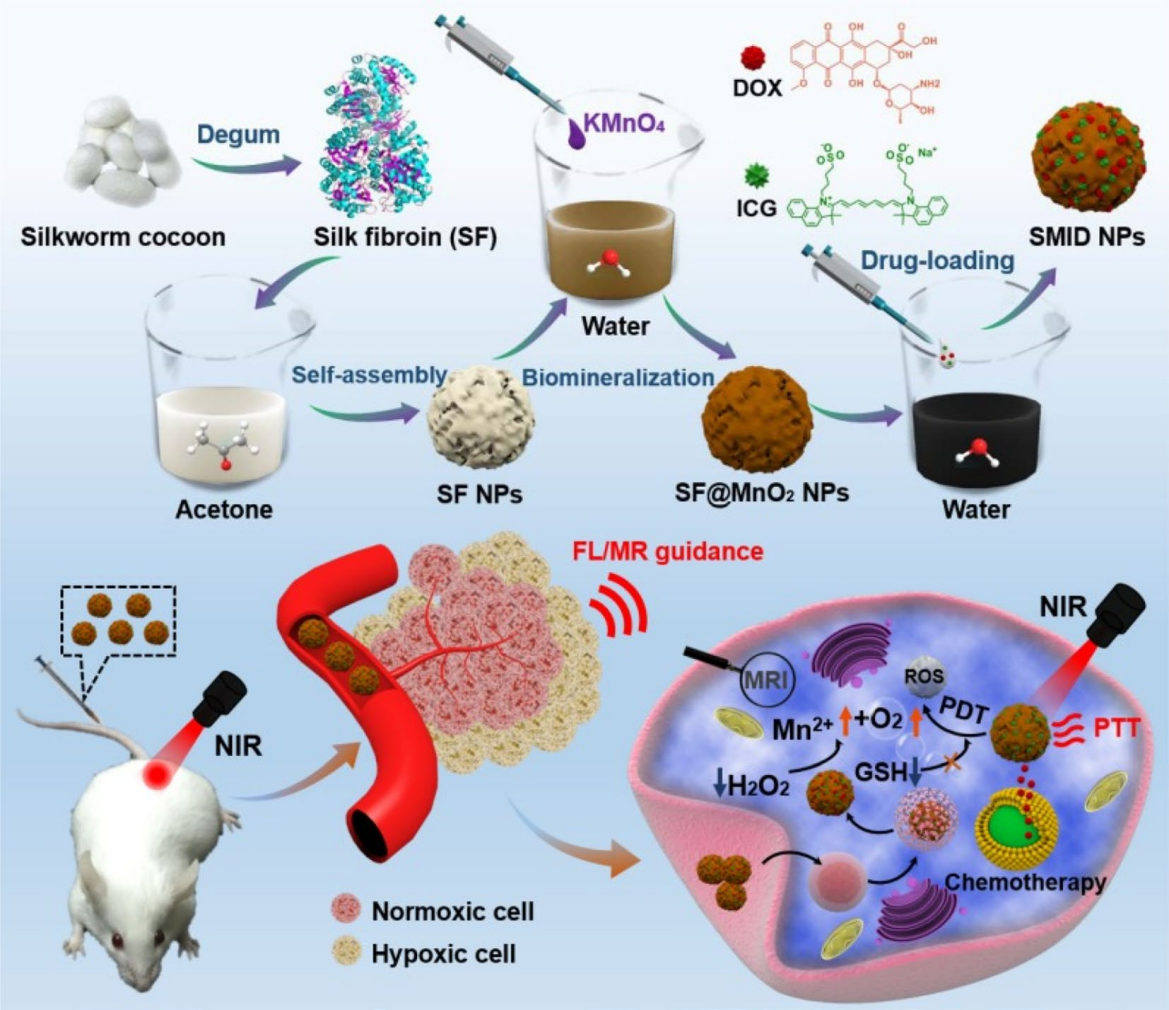
Schematic diagram of the synthesis, dual-model imaging, and combination therapy of multifunctional platform SMID. Upper: The silk fibroin (SF) is extracted from the silkworm cocoon and self-assembles into SF nanoparticles. The surface of SF nanoparticles is biomineralized by MnO_2_ (SF@MnO_2_). ICG and DOX are both loaded onto SF@MnO_2_ (SMID); Bottom: After intravenous injection, the tumor sites of mice are irradiated with a near-infrared (NIR) laser. The Mn^2+^ is produced under an acidic microenvironment, and ICG makes SMID an appropriate contrast agent for MR/fluorescence imaging. DOX and ICG is used for chemotherapy and photodynamic/photothermal therapy, respectively [166]

Yang et al. [166] used SF as a biotemplate and reductant to build a multifunctional SF@MnO_2_ platform (Fig. 4). DOX and photodynamic agent indocyanine green (ICG) can be co-loaded onto porous SF@MnO_2_ nanoparticles, producing a nanoparticle (SF@MnO_2_ loaded with ICG and DOX, termed SMID). MnO_2_ can act as a catalyst and promote the decomposition of endogenous hydrogen peroxide, producing oxygen to reduce tumor hypoxia and enhancing the effect of photodynamic therapy. Besides, MnO_2_ would decompose in response to a slightly acidic tumor environment, releasing DOX and producing Mn^2+^ that was a contrast agent to enhance the image contrast of T_1_-weighted MRI. Also, the temperature of the tumor site increased under laser irradiation because of ICG to achieve combined PTT/PDT/chemotherapy with the direction of MR/fluorescence dual-model imaging.

### Immunotherapy

There are many types of tumor immunotherapy, but most of them exert their anti-tumor effect through T cells in essence [167]. The immune checkpoint is a class of immunosuppressive molecules. Blocking immune checkpoints is one of the effective strategies to enhance T cell activation. It is also a popular target for anti-tumor drug development in recent years. Yang et al. [37] used ferritin nanocages to encapsulate ZnF_16_Pc, a kind of near-infrared photosensitizer, combining it with NLG_919_ (immune checkpoint IDO inhibitors) loaded poly (lactide-co-glycolic)-block-poly (ethylene glycol) (PEG-PLGA) nanoparticles covalently. This effect realized the codelivery of photosensitizer and an immune checkpoint inhibitor. The in vivo results showed that after in-situ injection of composite nanoparticles, drugs are released at an accelerated rate under acidic conditions. The consequent ROS and tumor-associated antigens inhibited tumor growth and increased T cell activity. This research combined PDT and immunotherapy and improved the efficacy of cancer therapy.

**Fig. 5:**
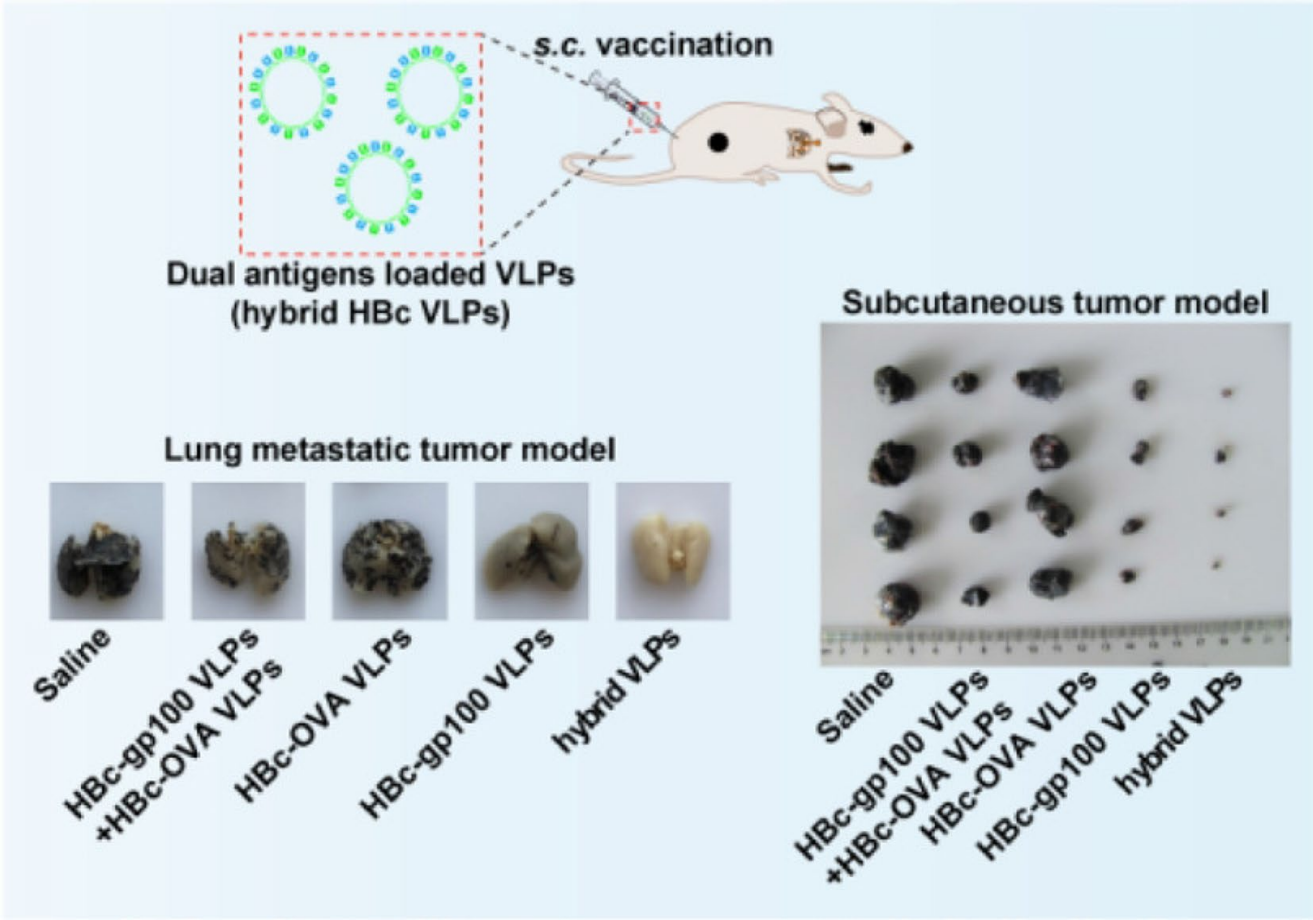
Schematic illustration of the therapy and the therapeutic effect of the tumor [168]. Upper: Schematic illustration of the construction of dual antigens loaded HBc VLP and the subcutaneous inoculation; Bottom left: Results of treatment of lung metastases. Compared to saline and single antigen-loaded VLPs, hybrid VLPs significantly inhibited the lung metastasis of melanoma; Bottom right: Results of treatment of subcutaneous melanoma. Compared to saline and single antigen-loaded VLPs, hybrid VLPs inhibited tumor growth and reduced the tumor volume to the maximum extent

Cheng et al. [168] fused pattern antigen OVA_257-264_ and specific antigen of melanoma gp100 into HBc based VLP through genetic recombination, constructing a novel dual antigen delivery system (Fig. 5). The hybrid VLP could accelerate the maturation of dendritic cells, induce a strong immune response in vivo and effectively inhibit the growth of subcutaneous melanoma and the formation of metastatic melanoma in the lung.

Schneider et al. [169] displayed tumor-associated antigens on the surface of VLP originated from murine leukemia virus. VLP displayed with the tumor-associated antigens triggered a powerful immune response due to the immunogenicity of VLP itself and effectively treated the tumor, showing the potential of VLP as a tumor vaccine.

### Clinical trials

The ClinicalTrials.gov website shows that albumin-based nanoparticles have been employed for treating cancer in clinical trials. Among them, Abraxane is the most common clinical drug to treat many kinds of cancer. We have summarized some typical clinical trials of the albumin-based nanoparticles applied for cancer therapy in Table 3.

**Table 3:** Clinical trials of protein nanoparticles applied for cancer therapy

Protein nanoparticles	Tumor type	Clinical studies	Year	Database (ID)
Albumin	Lung cancer	ABI-007 in Treating Patients with Chemotherapy-Naïve Stage IV Non-Small Cell Lung Cancer	2003–2008	ClinicalTrials.gov (NCT00077246)
Albumin	Ovarian cancer, fallopian tube cancer, and peritoneal cancer	Sargramostim and Paclitaxel Albumin-Stabilized Nanoparticle Formulation in Treating Patients with Advanced Ovarian Cancer, Fallopian Tube Cancer, or Primary Peritoneal Cancer That Did Not Respond to Previous Chemotherapy	2006–2011	ClinicalTrials.gov (NCT00466960)
Albumin	Solid tumors	Recombinant EphB4-HSA Fusion Protein with Standard Chemotherapy Regimens in Treating Patients with Advanced or Metastatic Solid Tumors	2015-	ClinicalTrials.gov (NCT02495896)
Albumin	Breast cancer	Romidepsin and Abraxane in Treating Patients with Metastatic Inflammatory Breast Cancer	2014–2016	ClinicalTrials.gov (NCT01938833)
Albumin	Pancreatic cancer	Gemcitabine, Nab-paclitaxel and KPT-330 in Advanced Pancreatic Cancer	2014-	ClinicalTrials.gov (NCT02178436)
Albumin	Melanoma	ABI-007 in Treating Patients with Inoperable Locally Recurrent or Metastatic Melanoma	2004–2010	ClinicalTrials.gov (NCT00081042)

## Protein nanoparticles as contrast agents’ carriers for cancer imaging

### Optical imaging

Optical imaging includes fluorescence imaging, bioluminescence imaging, and photoacoustic imaging. Protein nanoparticles can carry fluorescent dyes for in vivo fluorescence imaging. An et al. [170] constructed a Cy5 dye like small ultra-red fluorescent protein (smURFP), and combined it with BSA to fabricate a composite nanoparticle. This composite nanoparticle can be injected into tumor mice intravenously to target the tumor via the EPR effect while imaging the tumor in vivo simultaneously. In addition, this fluorescent probe solved the problem of poor tissue penetration of green fluorescence. Hu et al. [171, 172] developed the chitosan-modified silk sericin nanoparticles (SSC@NPs) and explored their redispersibility as well as biocompatibility in vivo. The in vitro results showed SSC@NPs had good colloidal stability without any dispersion stabilizer. The in vivo results showed that SSC@NPs gathered at the tumor through the EPR effect, and the loaded ICG could image the tumor.

**Fig. 6:**
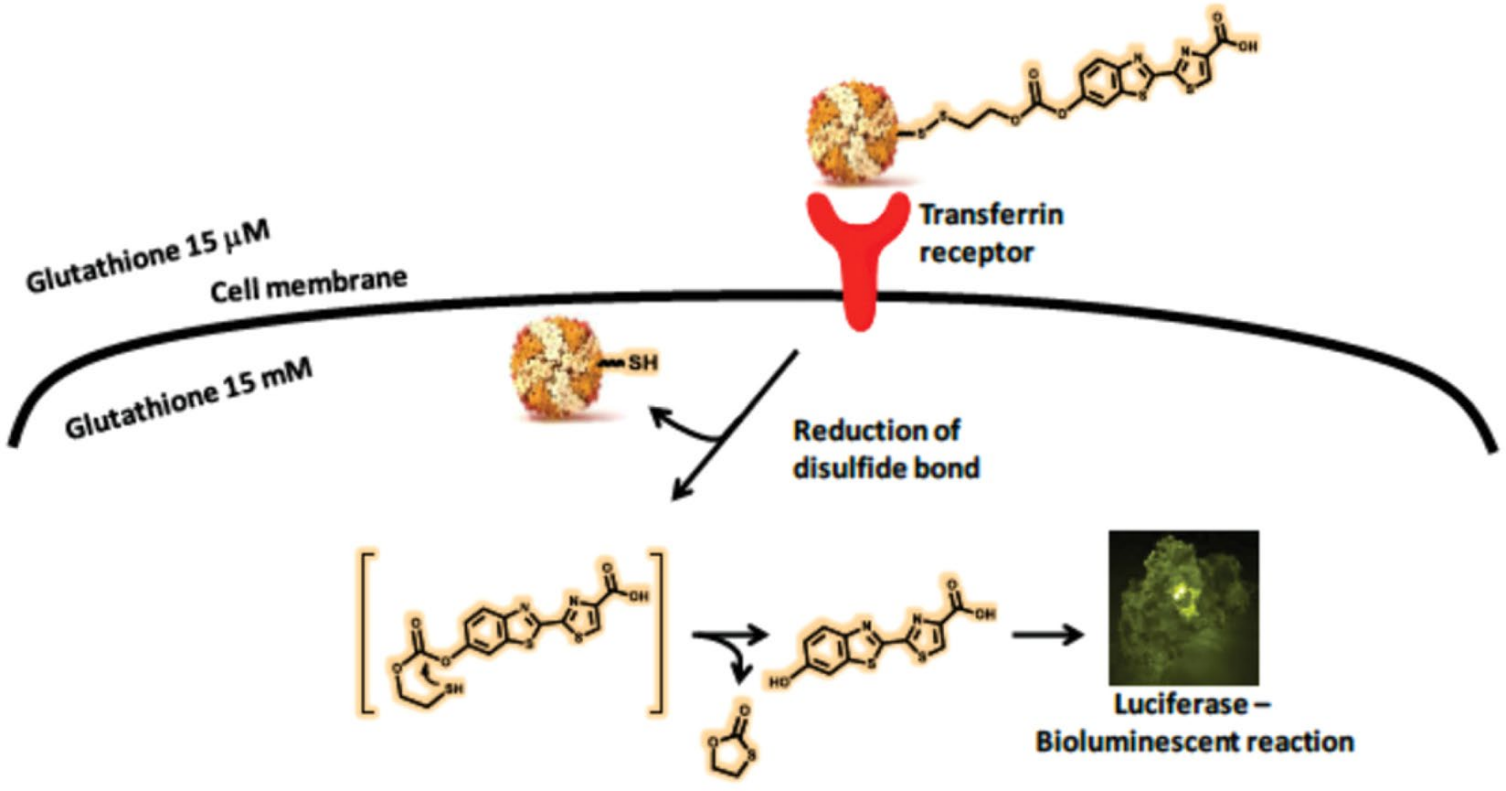
Schematic representation of the mechanism by which Luc-linker@HFn is recognized by the transferrin receptor and carries out bioluminescence. Luc-linker@HFn is internalized by cancer cells through TfR1. Intracellular GSH can break the disulfide bond and release free luciferin. Luciferin is able to react with luciferase and perform bioluminescence imaging [173]

H-ferritin (HFn) has a specific affinity to transferrin receptor 1 (TfR1). It is specifically overexpressed in the majority of cancers, so the protein nanoparticles can target tumors. Bellini et al. [173] used HFn to construct a kind of luciferin-loaded apoferritin nanoparticles (Luc-linker@HFn) (Fig. 6). The linker between HFn and luciferin was sensitive to GSH, so the complex could release luciferin in the cytoplasm of cancer cells and carried out bioluminescence imaging (BLI) of the luciferin-luciferase binding system. The bioluminescence imaging in vitro and in vivo could directly demonstrate that protein nanoparticles were internalized into cancer cells with high efficiency, diminishing the background signal. The composite nanoparticles are powerful imaging agents in luciferase tumor models.

Wang et al. [174] constructed copper sulfide (CuS) loaded ferritin nanocages (CuS–Fn) through a biomimetic synthesis method. Due to the good near-infrared absorbance of CuS–Fn, the nanoparticles could be applied in photoacoustic imaging (PAI) in vivo. When ^64^Cu was introduced, the nanoparticles could also act as a positron emission tomography (PET) imaging contrast agent. Therefore, PTT directed by dual-modal imaging was achieved, and the tumor was completely eliminated.

### Magnetic resonance imaging (MRI)

Protein nanoparticles usually perform MRI imaging by carrying magnetic nanoparticles or MRI contrast agents. Tao et al. [175] modified iron oxide nanoparticles with BSA and macromolecule (poly(acrylic acid)-poly(methacrylic acid), PMAA-PTTM) respectively to compare their MRI contrast performances. After intravenous injection of Fe_3_O_4_-BSA nanoparticles, in vivo T_1_-weighted MRI of the liver and kidney got darkened, indicating that Fe_3_O_4_-BSA was a more suitable T_2_-weighted contrast agent. However, the mice injected with Fe_3_O_4_-PMAA-PTTM showed the opposite result, suggesting that the surface ligands played an important role in MRI imaging. Mandal et al. [176] combined iron oxide and fluorescein-labeled monoclonal antibodies with collagen to construct a new type of collagen-based magnetic nanoparticle (CFeAb*D). The in vitro MRI images showed that after antibody treatment, more nanoparticles were endocytosed by gastric cancer cells through receptor-mediated endocytosis.

**Fig. 7:**
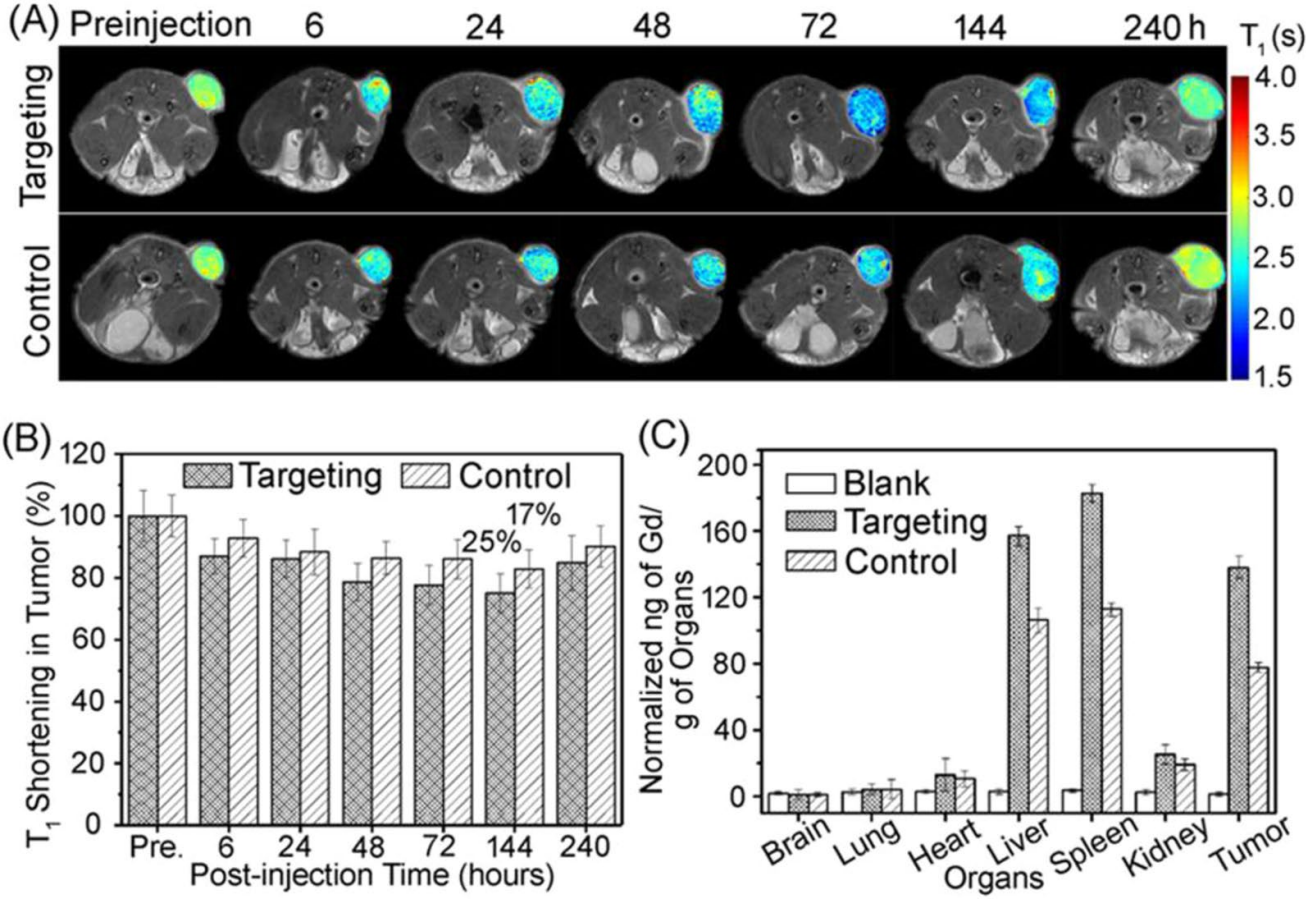
In vivo MRI results. **A** T1 weighted MR images of prostate cancer tumor-bearing mice at specific time points. **B** Quantitative analysis of T1 shortening in tumor. **C** Biodistribution of Gd complex in main organs and tumor (240 h post injection) [178]

Cai et al. [177] conjugated an MRI T1 contrast agent Gd-DTPA to the surface of H-ferritin nanoparticles. The T1 weight signal at the tumor site was significantly enhanced only 10 min after intravenous injection. Hu et al. [178] used the cavity of Physalis mottle virus (PhMV)-like nanoparticles to load Cy5.5 dye and Gd-DOTA to achieve fluorescence and MRI dual-model imaging. The targeting peptide conjugated on the surface of PhMV enabled the VLP to target the tumor. The in vivo MR imaging proved the ability of the composite particles to target tumors and achieve prolonged tumor retention (Fig. 7).

Ferritin can act as a MRI reporter gene as well. Lu et al. [179] found that overexpression of the ferritin reporter gene could further decrease the T2 signal at the tumor site. Also, Cohen et al. [180] confirmed that glioma cells transfected with ferritin also showed a strong signal on the T2 weighted images.

### Positron emission tomography (PET)

^64^Cu is a useful radioisotope for PET imaging. Lin et al. [83] structured a dual-modal imaging probe by combining near-infrared fluorescence imaging with PET imaging. The author firstly modified RGD4C peptide genetically and fluorescent dye Cy5.5 onto the surface of H-ferritin nanoparticles chemically. ^64^Cu was loaded through co-incubation with ^64^CuCl_2_ at pH = 2. When the pH was returned to 7.4, the ferritin could reconstruct nanostructures and achieved triple-loading. The in vivo PET and near-infrared fluorescence images showed that after tail vein injection of the ferritin probes, the probes could enrich at the tumor as a result of both RGD targeting peptide and EPR effect. Similarly, Aanei et al. [181] radiolabeled the genome-free capsid of bacteriophage MS2 with ^64^Cu and modified anti-EGFR antibodies to the surface of capsid to form a neoteric VLP. A whole day after injecting the VLP, PET/CT imaging of breast cancer tumor-bearing mice was obtained.

Xie et al. [182] also used HSA to encapsulate dopamine modified iron oxide, and labeled the nanoparticles with Cy5.5 and ^64^Cu-DOTA, which were contrast agents for fluorescence imaging and PET imaging respectively. Furthermore, iron oxide was an MRI imaging contrast agent. Therefore, the HSA nanoparticles are a new type of multifunctional theranostic platforms that could perform tri-modal imaging. The encapsulation of iron oxide particles into HSA matrices to form the nanosystems was a process similar to drug cargo loading which took full advantages of the HSA. The surface of the dopamine modified iron oxide nanoparticles was rich in amine groups. Compact HSA coating could be realized just by adsorbing due to the natural multivalency and good solubility of protein-based nanoparticles. The hydrodynamic size of the nanoparticles changed from 19.0 ± 0.9 nm to 29.4 ± 1.2 nm after the HSA coating. Cy5.5 and ^64^Cu-DOTA were labeled by coupling with lysines of HSA to get about twenty Cy5.5/DOTA molecules on the surface of each nanoparticle.

### Computed tomography (CT)

Although CT imaging is often used in combination with PET, it can act as an independent imaging modality [183]. Chu et al. [184] used BSA as a biotemplate to make ultrasmall Au–Ag@BSA bimetallic protein nanoparticles with an average size of 2–4 nm. They manipulated the ratio of gold to silver in the synthesis of nanoparticles. Interestingly, they found that when the ratio of gold to silver is 3: 2, the protein particles displayed great CT imaging performance. Besides, the particles showed good biocompatibility both in vitro and in vivo.

**Fig. 8:**
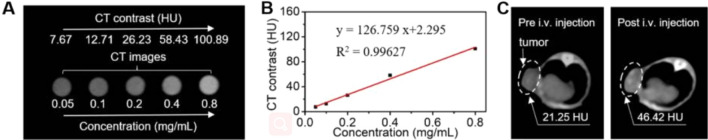
In vitro and in vivo CT imaging results. **A** CT imaging of Dox@AFBS solutions with different concentrations in vitro. **B** Contrast and concentration curves for Dox@AFBS. **C** CT imaging of Hela tumor-bearing mice before and after intravenous injection of Dox@AFBS in vivo [185]

Zhang and her coworkers [185] employed apoferritin to construct a novel nano-diagnosis and treatment platform; DOX was encapsulated inside the ferritin cavity, and radiation sensitizer bismuth sulfide (Bi_2_S_3_) crystals were embedded in the protein shell (Dox@AFBS). Bismuth sulfide can also be used as a radiation sensitizer because Bi^3+^ has a large attenuation coefficient of X-ray. The CT imaging showed that Dox@AFBS accumulated at the tumor site after injection (Fig. 8). These studies demonstrated that the combination of chemotherapy and radiotherapy could effectively diminish the volume of subcutaneous Hela tumor.

## Conclusions and outlook

As drug carriers for cancer diagnosis and treatment, protein nanoparticles have the advantages of biocompatibility and biodegradability compared with other carriers such as inorganic nanoparticles. Facile functionalization of protein surfaces makes it easy to load drugs, targeting ligands or other functional small molecules onto them, thus constructing multi-functional theranostic platforms. Hence, we summarize the preparation methods of several most common protein nanoparticles and the latest advances of their use in cancer treatment and imaging.

However, there are several problems with protein nanoparticles: (1) some proteins are not that stable, and their structures may change during synthesis of the nanoparticles; (2) some protein nanoparticles are not uniform in size and size adjustment during preparation is not as easy as inorganic nanoparticles [186]; (3) the mechanism of protein nanoparticles mediated transport is unclear [187]. Besides, most nanoparticles for tumor therapy in the clinical stage are liposome or lipid nanoparticles, and there are few protein nanoparticle based clinical drugs [188]. As with other nanoparticles, there are issues of circulation time, biological distribution and mass production [27, 186]. Protein nanoparticles also face the problem of the low drug cargo loading rate. Protein nanoparticles should not be conjugated with ligands of a complex structures in cases when such conjugation may affect the self-assembly of protein nanoparticles themselves. In addition, nanoparticles smaller than 20 nm or larger than 250 nm were easily cleared by the kidney or the reticuloendothelial system (RES). Lacking a long circulation time reduces the effectiveness of the treatment [189, 190]. Researchers urgently need to address the issue of drug loading, long-time tumor retention, and mass production of protein nanoparticles. Anyway, the successful preparation of Abraxane® (paclitaxel loaded albumin nanoparticle) is uplifting. It is believed that with the continuous efforts of researchers, protein nanoparticles will play a greater part in the field of cancer diagnosis and treatment.

## Data Availability

The datasets used and analysed during the current study are available from the corresponding author on reasonable request.

## Abbreviations

GC–MS: Gas chromatography/mass spectrometry
NGS: Next-generation sequencing
ELISA: Enzyme-linked immunosorbent assay
MDR: Multidrug resistance
VLPs: Virus-like particles
OVA: Ovalbumin
BSA: Bovine serum albumin
HSA: Human serum albumin
EDC: *N*-(3-Dimethylaminopropyl)-*N*’-ethylcarbodiimide hydrochloride
NHS: *N*-Hydroxysuccinimide
O/W: Oil-in-water
CP: Coat protein
SP: Scaffolding protein
DOX: Doxorubicin
Fn: Ferritin
SF: Silk fibroin
SS: Silk sericin
HAp: Hydroxyapatite
EPR: Enhanced permeability and retention
CPP: Cell penetrating peptide
FAP: Fibroblast activation protein
scFv: Single-chain variable fragment
HBc: Hepatitis B core
GSH: Glutathione
PAA: Polyacrylic acid
RSF: Regenerated silk fibroin
PTT: Photothermal therapy
PDT: Photodynamic therapy
AFN: Apoferritin
PTX: Paclitaxel
AuNPs: Gold nanoparticles
SRF: Sorafenib
GPX4: Glutathione peroxidase 4
ICG: Indocyanine green
PEG-PLGA: Poly (lactide-co-glycolic)-block-poly (ethylene glycol)
PD-L1: Programmed death ligand 1
sgRNA: Single guide RNA
RNP: Ribonucleoprotein
TfR1: Transferrin receptor 1
BLI: Bioluminescence imaging
PAI: Photoacoustic imaging
PET: Positron emission tomography
MRI: Magnetic resonance imaging
CT: Computed tomography
PMAA-PTTM: Poly(acrylic acid)-poly(methacrylic acid)
PhMV: Physalis mottle virus
PNs: Protein nanoparticles
RES: Reticuloendothelial system
